# Extremely stringent activation of p16^INK4a^ prevents immortalization of uterine cervical epithelial cells without human papillomavirus oncogene expression

**DOI:** 10.18632/oncotarget.10120

**Published:** 2016-06-17

**Authors:** Su Hang, Agnes F.Y. Tiwari, Hextan Y.S. Ngan, Yim-Ling Yip, Annie L.M. Cheung, Sai Wah Tsao, Wen Deng

**Affiliations:** ^1^ School of Nursing, Li Ka Shing Faculty of Medicine, The University of Hong Kong, Hong Kong, SAR, China; ^2^ College of Forensic Sciences, Xi'an Jiaotong University Health Science Center, Xi'an, Shanxi Province, P.R. China; ^3^ Department of Obstetrics and Gynaecology, The University of Hong Kong, Hong Kong, SAR, China; ^4^ School of Biomedical Sciences, Li Ka Shing Faculty of Medicine, The University of Hong Kong, Hong Kong, SAR, China

**Keywords:** cervical epithelial cells, immortalization, p16^INK4a^, telomerase, HPV-negative

## Abstract

Cervical epithelial cell immortalization with defined genetic factors without viral oncogenes has never been reported. Here we report that HPV-negative cervical epithelial cells failed to be immortalized by telomerase activation or the combination of p53 knockdown and telomerase activation. Under those conditions, p16^INK4a^ expression was always elevated during the late stage of limited cell lifespan, suggesting that cervical epithelial cells possess an intrinsic property of uniquely stringent activation of p16^INK4a^, which may offer an explanation for the rarity of HPV-negative cervical cancer. Combining p16^INK4a^ knockdown with telomerase activation resulted in efficient immortalization of HPV-negative cervical epithelial cells under ordinary culture conditions. Compared with the HPV16-E6E7-immortalized cell lines derived from the same primary cell sources, the novel HPV-negative immortalized cell lines had lower degrees of chromosomal instability, maintained more sensitive p53/p21 response to DNA damage, exhibited more stringent G2 checkpoint function, and were more resistant to replication-stress-induced genomic instability. The newly immortalized HPV-negative cervical epithelial cell lines were non-tumorigenic in nude mice. The cell lines can be used not only as much-needed HPV-negative non-malignant cell models but also as starting models that can be genetically manipulated in a stepwise fashion to investigate the roles of defined genetic alterations in the development of HPV-negative cervical cancer.

## INTRODUCTION

Uterine cervical cancer is one of the most frequently occurring cancers in women worldwide [1]. Infection with high-risk human papillomavirus (HPV) such as HPV16 or HPV18 has been identified as a crucial etiological factor of at least 90% of cervical cancers [2]. However, the mechanisms for the development of HPV-negative cervical cancers still remain very poorly understood. The well-known oncogenic functions of high-risk HPV include the deregulated expression of viral oncogenes E6 and E7, which inactivate the master tumor suppressors, p53 and Rb proteins, respectively, by accelerating proteolytic degradation [3]. In addition, high-risk HPV E6 can also activate telomerase in epithelial cells, and interact with PDZ proteins (postsynaptic density signaling proteins, *Drosophila* disc large proteins and zonula occludens 1 proteins) that play critical roles in a variety of cellular and molecular processes including those important for cell polarity and signal transduction [4].

Cellular immortalization is an early and indispensible step for cancer development and has been regarded as a hallmark of cancer [5]. To date, most immortalized cervical epithelial cell lines were established by high-risk HPV infection or E6/E7 expression [e.g, 6–11]. Although a Rho kinase inhibitor enabled immortalization of human cervical epithelial cells without the expression of viral oncogenes, the immortalization needs the continuous presence of fibroblast feeder cells [12]. Immortalization of human cervical epithelial cells without feeder fibroblasts and viral oncogenes has never been reported so far.

With the application of HPV vaccines, the relative ratio of HPV-negative to HPV-positive cervical cancer may increase in the future because of the decrease in incidence of HPV-induced cervical cancer. The establishment of immortalized HPV-negative cervical epithelial cell lines may have important applications in the illustration of stepwise events leading to HPV-negative cervical cancer and in development of targeted therapy.

Immortalization of human somatic cells requires telomere maintenance, either by telomerase activation, or in some rare cases by alternative telomere lengthening mechanism. Telomerase activation is commonly achieved by overexpression of hTERT, the catalytic subunit of telomerase. However, up to date, it is still controversial whether telomerase activation alone is sufficient for immortalization of human epithelial cells or the requirements are cell-type/context dependent. Although some reports have defined that both inactivation of p16^INK4a^/Rb pathway and telomerase activation are necessary and sufficient for immortalization of examined epithelial cell types [13–15], others reported that inactivation of p16^INK4a^ was not required for immortalization of cells expressing hTERT as long as the cells were cultured with the fibroblast feeder layer [16]. Interestingly, the group led by Rheinwald, the initial inventor of fibroblast feeder layer system, demonstrated that even under the condition of the fibroblast feeder layer, keratinocytes still needed the inactivation of p16^INK4a^ and p53 to achieve immortalization [17]. In contrast, an esophageal epithelial cell line and a pancreatic duct epithelial cell line could be immortalized by hTERT expression alone, without the inactivation of p16^INK4a^ and without using fibroblast feeder layer [18, 19]. Furthermore, data from multiple cell sources showed that there were intrinsic differences in the basal levels of p16^INK4a^ expression [20]. The cell strains with low basal levels of p16^INK4a^ were insensitive to further p16^INK4a^ activation, and the senescence in those cell strains could be reversed by suppression of telomere-shortening-triggered senescence signaling, such as by p53 suppression or hTERT overexpression; whereas in those cell strains sensitive to p16^INK4^ activation, the senescence state remained irreversible by p53 suppression and telomerase activation [20]. This finding offers an explanation why some cell strains can be directly immortalized by telomerase activation alone whereas others cannot. Importantly, many cell-types such as esophageal, mammary, nasopharyngeal, prostate, retina pigment epithelial cells, oral keratinocytes, foreskin keratinocytes, adenoid epithelial cells, endothelial cells, and some strains of human fibroblasts exhibited spontaneous loss of p16^INK4a^ expression by p16^INK4a^ gene deletion or promoter methylation during prolonged culture of hTERT-expressing cells which initially expressed high levels of p16^INK4a^ prior to immortalization [13, 15, 20–28]. Given the fact that human cervical cancer are unique in that they are highly dependent upon persistent high-risk HPV infection, we sought to explore whether HPV-negative cervical epithelial cells are intrinsically different from those reported cell types in the property of persistent activation of p16^INK4a^ expression that hinders immortalization without viral oncogene expression.

Our continuous effort in the last 5 years demonstrated that HPV-negative cervical epithelial cells failed to be immortalized no matter hTERT was overexpressed alone or p53 expression was suppressed in combination with hTERT overexpression. Under those conditions, p16^INK4a^ expression was always increased during the late stage of limited cell lifespan. The combination of p16^INK4a^ knockdown with hTERT overexpression resulted in efficient immortalization of HPV-negative cervical epithelial cells under ordinary culture conditions. These findings strongly suggest that cervical epithelial cells possess an intrinsic property of uniquely stringent maintenance of p16^INK4a^ expression, which acts as a giant barrier to immortalization without HPV oncogene expression. We further found that, when compared with the cell lines immortalized by expressing HPV16 E6E7, the HPV-negative immortalized cervical epithelial cell lines were genetically more stable, had more sensitive response to DNA damage, exhibited more stringent G2 checkpoint function, and were more resistant to replication-stress-induced genomic instability. The HPV-negative immortalized cervical epithelial cell lines were non-tumorigenic in nude mice. Revelation of genetic elements for immortalization of HPV-negative cervical epithelial cells has important implications for the pre-cancer stage of poorly understood HPV-negative cervical neoplastic transformation.

## RESULTS

### Human uterine cervical epithelial cells were unable to be immortalized by hTERT overexpression alone

**Figure 1:**
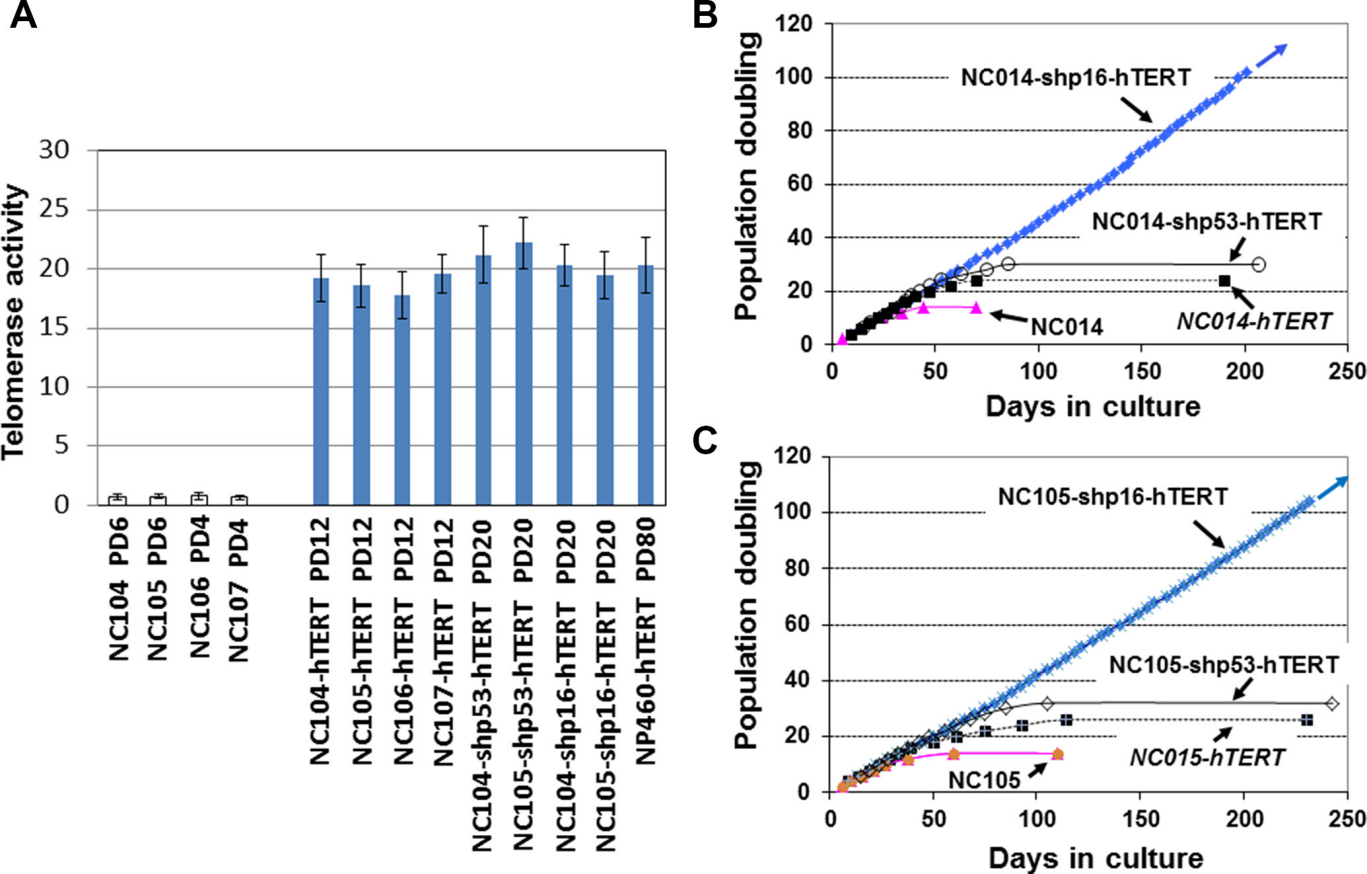
Telomerase and growth curves of cervical epithelial cells after introduction of different genetic factors (**A**) Telomerase activity (relative to “low activity” controls provided by the manufacturer) quantified according to protocols of TeloTAGGG Telomerase PCR ELISA kits. Error bars indicate standard deviations from 3 duplicates. (**B** and **C**) Growth curves of cervical epithelial cells with or without introduction of different genetic factors.

We have been involved in immortalization of multiple types of epithelial cells using various genetic elements [11, 26, 27, 29, 30], and have immortalized multiple esophageal and nasopharyngeal epithelial cell lines by overexpression of hTERT alone without using feeder fibroblasts [26, 27, 30]. Of note, those hTERT-immortalized cell lines were established after the cells acquired spontaneous loss or expression of low levels of p16^INK4a^. We therefore tested whether cervical epithelial cells had the same capacity of immortalization by overexpression of hTERT alone without using feeder fibroblasts. The primary cell sources (designated as NC104, NC105, NC106 and NC107) were cultured from normal uterine cervical tissues donated by 4 independent individuals. Without introduction of any ectopic genetic factors, the four parental cell strains had a maximum lifespan of 14 population doublings (PD) after the first passage in culture. We then used retroviral vectors expressing hTERT to activate telomerase in the four cell strains at PDs 4–8 when the cells were at the stage of active proliferation (the same method as we used before for esophageal and nasopharyngeal epithelial cell immortalization [26, 27, 30]). Telomerase activity was quantified by using enzyme-linked immunosorbent assay-based telomeric repeat amplification protocol (ELISA-TRAP). We confirmed that telomerase was successfully activated by hTERT overexpression compared with primary proliferating cells (Figure 1A). The levels of activated telomerase activity in all four hTERT-expressing cervical cell strains reached at the similar level with our previously immortalized nasopharyngeal epithelial cell line by hTERT overexpression (NP460-hTERT). For the last 5 years, we have cultured an average of twenty T-75 flasks of hTERT-expressing cervical epithelial cells for each of the four cell strains (∼ 1 × 10^7^ cells in each flask resistant to drug selection for hTERT overexpression). Although lifespans of the cells were extended by limited population doublings, none of the cultures resulted in immortalization (Table 1). In each culture flask, the cells eventually entered into a plateau stage, during which the cell numbers stopped increasing, and eventually all cells acquired enlarged cell sizes, the typical morphology of cellular senescence. We cultured the cells at senescence in flasks as long as possible (for an average of 4 months) with fresh medium replacement every 3 days to see if some rare cells could acquire proliferative capacity. However, eventually all of the cells became detached from the flasks and died out. As examples, the typical growth curves of NC104-hTERT and NC105-hTERT are shown in Figure 1B and 1C. Compared with our previous success in hTERT-mediated immortalization of other types of epithelial cells [26, 27, 30], our five years of continuous effort on multiple cervical epithelial cell strains led us to conclude that human cervical epithelial cells are generally unable to be immortalized by hTERT overexpression alone.

**Table 1:** Attempts of immortalization of cervical epithelial cells by hTERT overexpression

Cell strain	Number of flasks cultured	Number of immortalized clones	Average lifespan (population doubling ± SD)*
NC104-hTERT	23	0	24 ± 2
NC105-hTERT	22	0	26 ± 2
NC106-hTERT	18	0	20 ± 1
NC107-hTERT	20	0	22 ± 2

* Standard deviatation.

### Persistent increase in p16^INK4a^ expression in hTERT-expressing cervical epithelial cells at late stage of cell lifespan

**Figure 2:**
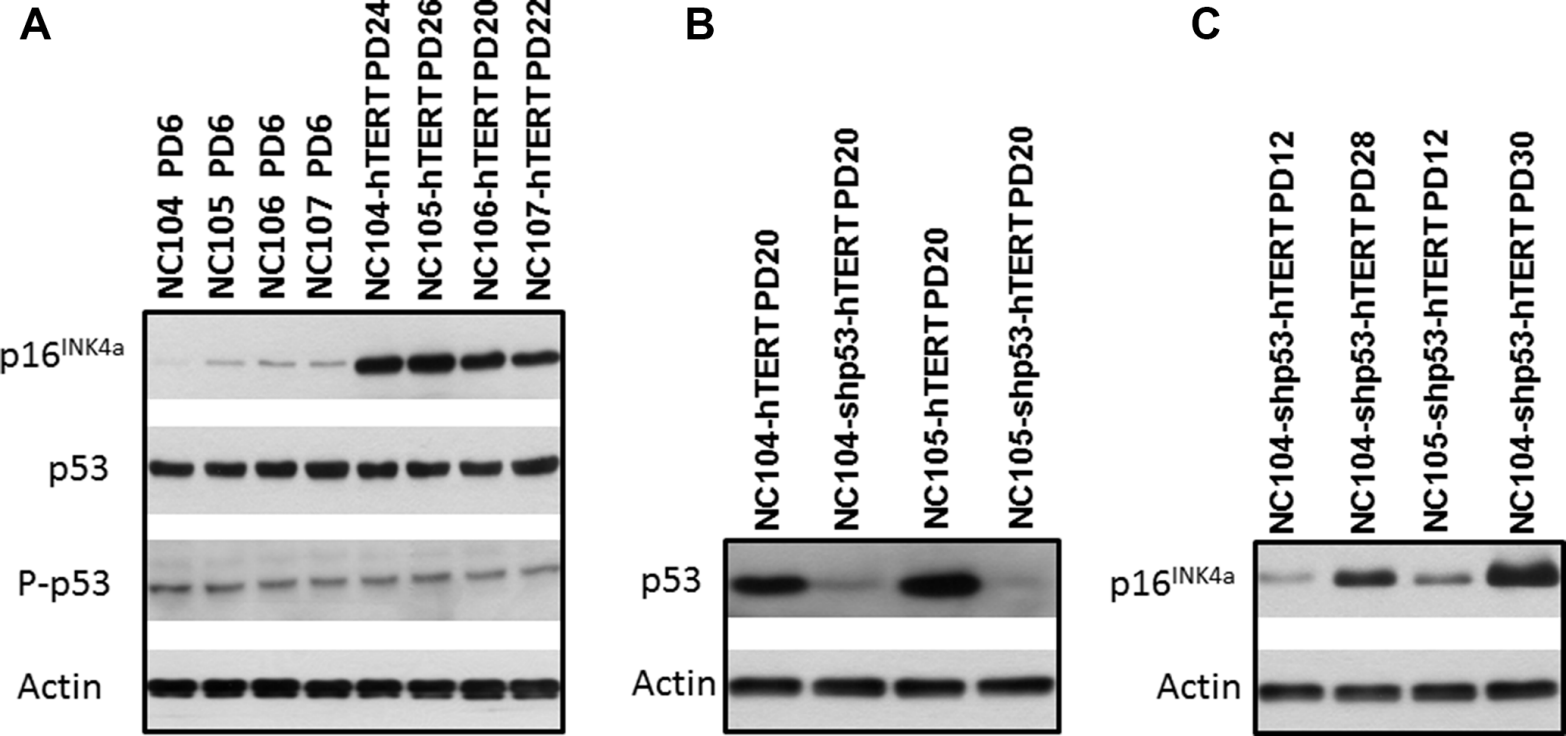
Western blotting analysis for protein expression of p16^INK4a^, p53, and actin that served as protein loading controls

It has been shown that there are intrinsic differences among some cell types or cell strains in the ability to induce p16^INK4a^ expression at senescence [20]. The increase in p16^INK4a^ expression is believed to be a sensitive response to stress *in vitro* as well as *in vivo*. After the cells enter the p16^INK4a^/Rb-mediated senescence, neither ectopic p53 inactivation nor subsequent p16^INK4a^/Rb inactivation combined with hTERT overexpression can rescue the senescence phenotype [20]. This correlates with the finding that an irreversible repressive heterochromatin state is established by p16^INK4a^/Rb activation [31]. However, in many cell types [13, 15, 20–28], some hTERT-expressing cells expressed low levels of p16^INK4a^ or lost p16^INK4a^ expression, and eventually became dominant in culture, leading to immortalization. Yet our Western Blotting analysis consistently revealed sharp increases in the protein expression of p16^INK4a^ in all cultures of hTERT-expressing cervical epithelial cells at the late stage of senescence compared with that at the early pre-senescence stage, as exemplified by the typical data in Figure 2A. The expression levels of total p53 and phosphorylated p53 (at Ser- 15, the active form), did not showed remarkable differences between the early and the late stage of the cells' lifespan (Figure 2A). Therefore we conclude that cervical epithelial cells have an intrinsic property of stringent maintenance of p16^INK4a^ activation at senescence. This is consistent with our observation that there has been no any immortalized clone of hTERT-expressing cervical epithelial cells that could emerge from senescence in a total of 83 cultures of 4 cell strains (Table 1).

### p53 suppression plus hTERT expression was unable to immortalize cervical epithelial cells

The suppression of p53 in combination with hTERT expression has been reported to be able to immortalize ovarian surface epithelial cells [32]. It is of interest to test whether the similar finding could be obtained in cervical epithelial cells. We performed p53 knockdown using retroviral vectors expressing short hairpin RNA against p53 to inactivate p53 (the same vectors as for ovarian surface epithelial cell immortalization [32]) in combination with hTERT expression in NC104 and NC105 cells. The successful suppression of p53 expression was confirmed by Western Blotting analysis (Figure 2B). Strong telomerase activity was also detected (Figure 1A). However, p53 suppression plus hTERT overexpression failed to induce immortalization in both cervical epithelial cell strains. Actually, the lifespans (population doublings) of the cells were not remarkably extended by the combination of p53 knockdown with hTERT expression compared with the cells expressing hTERT alone (Figure 1B and 1C). Of note, the cells with p53 knockdown and hTERT expression exhibited increased levels of p16^INK4a^ at the late stage of their lifespan compared with their early lifespan (Figure 2C).

### Efficient immortalization of cervical epithelial cells by knockdown of p16^INK4a^ in combination with hTERT overexpression

**Figure 3:**
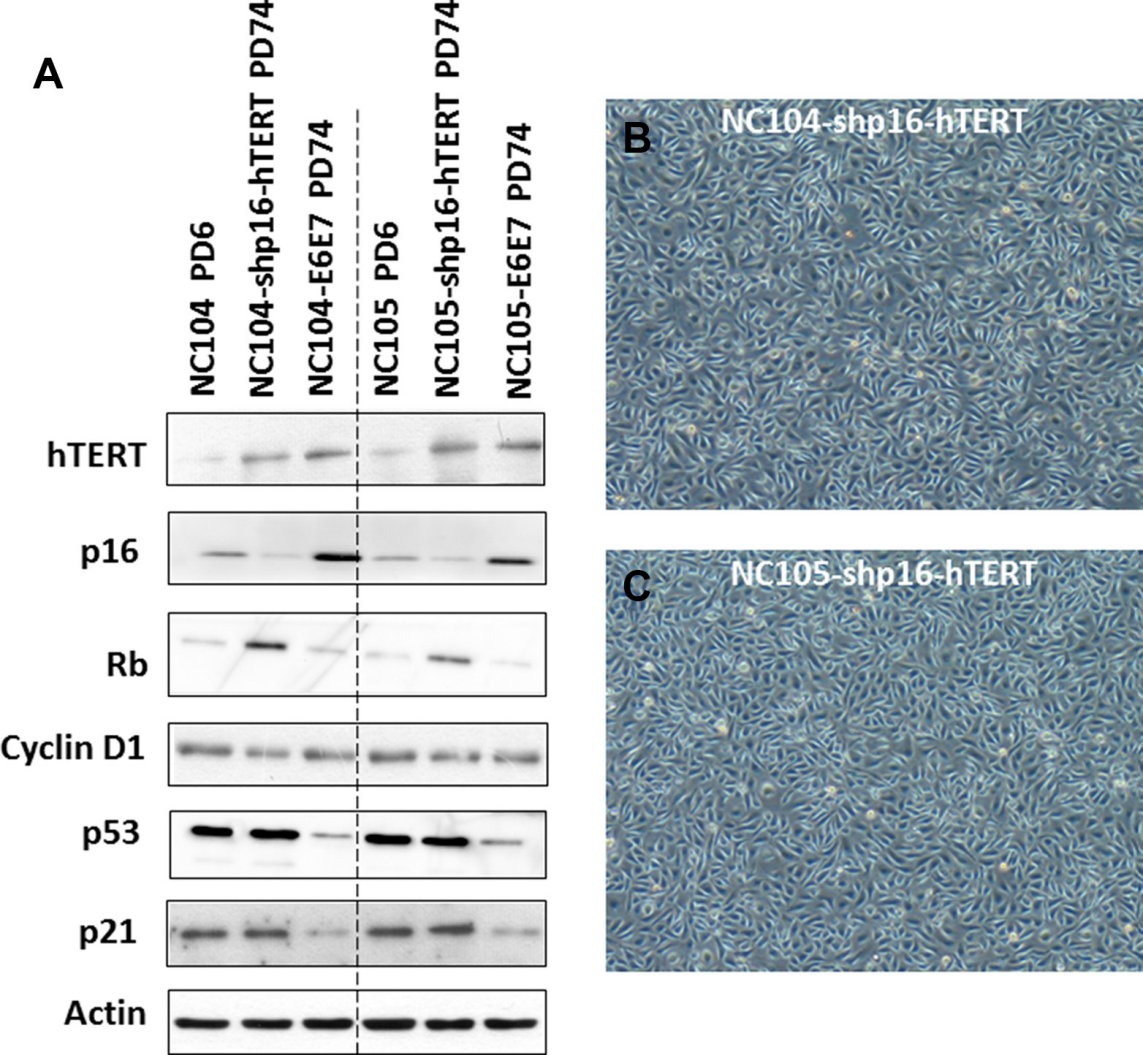
Protein expression of immortalization-related genes and morphology of immortalized cervical epithelial cells (A) Western blotting analysis for protein expression of hTERT and cell cycle regulation genes in primary, shp16-hTERT-immortalized, and HPV16-E6E7-immortalized cervical epithelial cells. (B and C) Typical morphology of immortalized cervical epithelial cells.

To test whether p16^INK4a^ activation truly functions as the dominant barrier to immortalization of cervical epithelial cells, we infected the proliferative cervical epithelial cells (NC104 and NC105) at PD 4 with lentiviral vectors expressing short hairpin RNA against p16^INK4a^ (shp16) to suppress p16^INK4a^ expression [20] and retroviral vectors expressing hTERT. This combination resulted in vigorous continuous cell proliferation far beyond the original lifespan of the parental cells. So far, the two cell lines, designated as NC104-shp16-hTERT and NC105-shp16-hTERT, have undergone over 100 PD without any signs of crisis or a slow growth phase, as shown by growth curves in Figure 1B and 1C. Because the lifespans have been extended for more than 50 PD beyond the original lifespans without any signs of slowing down in growth rates, the two novel cell lines are regarded as immortalized according to the published criteria [17]. The increased hTERT protein expression, the decreased p16 expression (Figure 3A), and the strong telomerase activity was confirmed in both shp16-hTERT-immortalized cell lines as compared with their parental cells (Figure 1A). Short tandem repeat (STR) analysis at eighteen loci showed that NC104-shp16-hTERT and NC105-shp16-hTERT cell lines had distinct STR profiles, confirming that they were derived from independent cell sources (Supplementary information, Supplementary Figures S1 and S2). As expected, identical STR profiles were found in our newly established NC104-shp16-hTERT cell line and the previously immortalized NC104-E6E7 cell line [11] because they were established from the same parental cell source. The same is true in NC105-shp16-hTERT and NC105-E6E7 cell lines (Supplementary Figures S1 and S2). Our RT-PCR analysis demonstrated that both NC104-shp16-hTERT and NC105-shp16-hTERT cell lines did not express HPV E6 and E7 genes, in contrast to NC104-E6E7 and NC105-E6E7 cell lines that were previously immortalized by HPV16 E6E7 (Supplementary Figure S3). These results clearly demonstrate that the maintenance of extremely stringent p16^INK4a^ activation is indeed a crucial barrier to immortalization of HPV-negative cervical epithelial cells.

### The newly immortalized cervical epithelial cell lines retained typical cytokeratin characteristic of cervical epithelial cells

**Figure 4:**
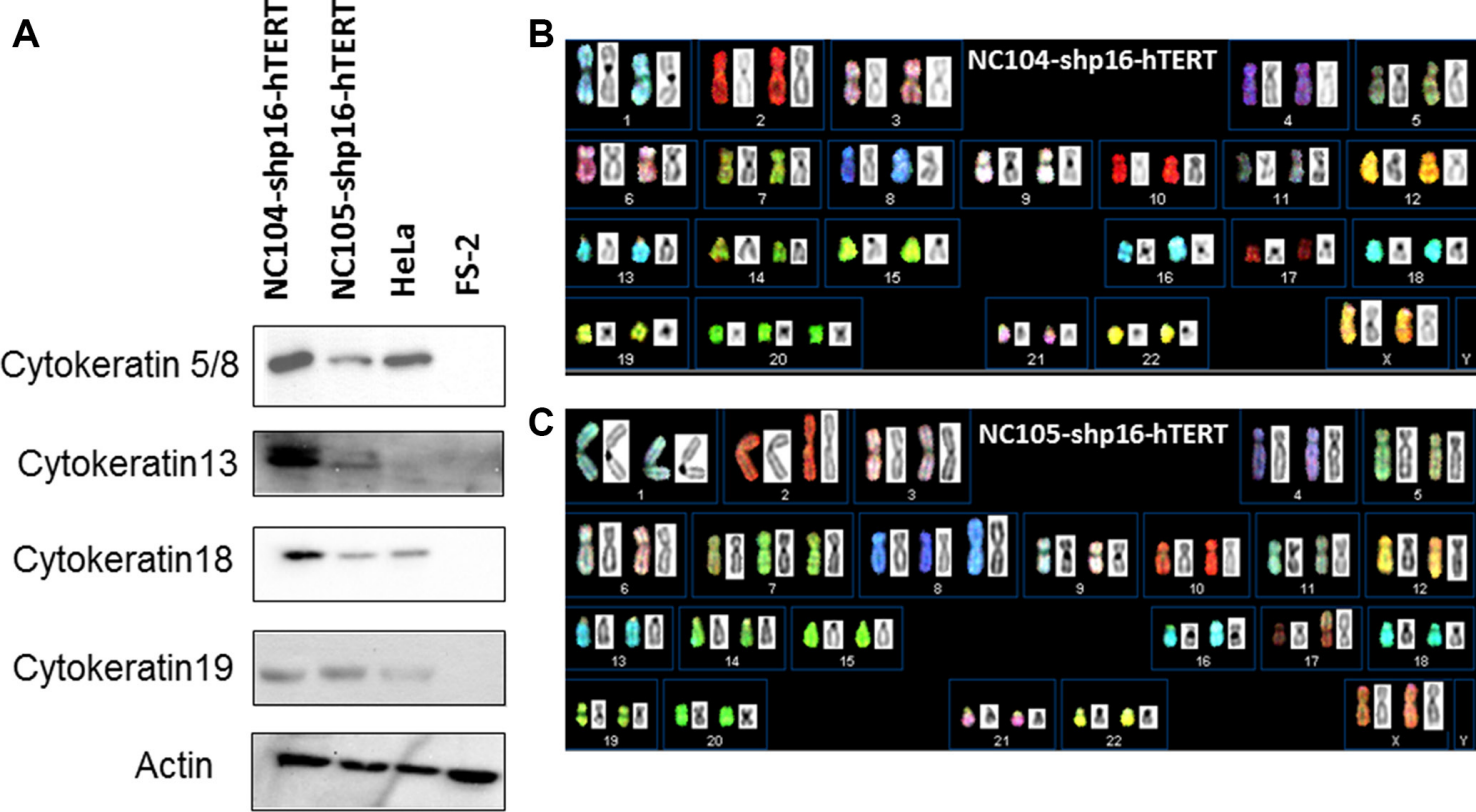
Cytokeratin expression and spectral karyotyping (SKY) analyses of immortalized cell lines (**A**) Protein expression of typical cervical epithelial cytokeratins in the immortalized cervical epithelial cell lines. HeLa and FS-2 cell lines served as positive and negative controls. (**B** and **C**) Typical SKY in the newly immortalized cervical epithelial cell lines. Karyotyping descriptions in shp16-hTERT and HPV16-E6E7-immortalized cell lines were presented in Table 2.

The immortalized NC104-shp16-hTERT and NC105-shp16-hTERT cell lines showed typical cuboidal morphology, which is characteristic of proliferative epithelial cells (Figure 3B and 3C). Western Blotting analysis was carried out to detect the expression of some typical epithelial cell cytokeratins. Cytokeratin 5/8, 13, 18 and 19 were detected in NC104-shp16-hTERT and NC105-shp16-hTERT cell lines (Figure 4A). The expression pattern is in agreement with the cytokeratin expressions in previously reported high-risk-HPV- or E6E7-immortalized cervical epithelial cell lines [6, 10]. As a control for our analysis, HeLa cancer cell line expressed cytokeratin 5/8, 18 and 19, but not cytokeratin 13, in consistence with the reported results in HeLa cell line [10]. In contrast, the cytokeratin expression was not detected in the fibroblast cell line FS-2, which served as a negative control.

### Alterations in gene expressions in p16^INK4a^/Rb and p53/p21 pathways

As mentioned above, our Western Blotting analysis demonstrated that p16^INK4a^ expression was successfully knocked down in NC104-shp16-hTERT and NC105-shp16-hTERT cell lines compared with their respective proliferative parental cells (Figure 3A). For comparison, the HPV16 E6E7-immortalized cell lines expressed higher levels of p16^INK4a^ protein than parental cells, in agreement with previous findings in other HPV- or E6E7-immortalized cell lines [33, 34]. The increased p16^INK4a^ expression in E6E7-expressing cells could be explained by the negative regulation of p16^INK4a^ by Rb [35]. Interestingly, the shp16-hTERT-immortalized cervical epithelial cell lines expressed higher levels of Rb than respective proliferative parental cells (Figure 3A). This is similar with the observation in hTERT-immortalized foreskin and adenoid keratinocytes accompanied with spontaneous loss of p16^INK4a^ expression [21]. Although cyclin D1 is a known downstream target of p16^INK4a^/Rb pathway, we observed no obvious change in the expression levels of cyclin D1 in the shp16-hTERT-immortalized cell lines, indicating the complex regulation of cyclin D1 [21]. There were no significant changes in expression levels of p53 and p21 after shp16-hTERT-mediated immortalization when compared with those in parental cells. This is in line with our result that the suppression of p53 is not required for immortalization of cervical epithelial cells once p16^INK4a^ expression was suppressed and telomerase was activated. For comparison, the clearly lower levels of p53 and p21 expression were detected in HPV16-E6E7-immortalized cell lines when compared with parental cells (Figure 3A).

### Karyotypic analysis

Twenty-four color spectral karyotyping (SKY) was performed to analyze whole-genome chromosomal abnormalities of the shp16-hTERT-immortalized cervical epithelial cell lines and their parental cells. At an early population doubling (PD 30), a normal karyotype was observed in the majority of shp16-hTERT-immortalized cells, and trisomy 20 or trisomy 7 was detected as a minor clone in NC104-shp16-hTERT and NC105-shp16-hTERT cell lines, respectively (Table 2). At PD80, a stable near-diploidy with trisomy 20 was observed in the majority (∼ 80%) of NC104-shp16-hTERT cells (Figure 4B and Table 2). In NC105-shp16-hTERT cells, a stable near-diploidy containing trisomy 7, an extra iso-chromosome composed of 8q, and a derivative chromosome 17, was detected in ∼ 90% of metaphases at PD 80 (Figure 4C and Table 2). Parental NC104 and NC105 cells at PD 4 had normal diploid karyotype. Therefore, the clonal chromosomal alterations in the immortalized cell lines were acquired during immortalization. Compared with clonal chromosomal aberrations in previously immortalized NC104-E6E7 and NC105-E6E7 cell lines at the same PD (Table 2), the shp16-hTERT-immortalized cells had fewer clonal aberrations, in particular, fewer clonal numerical abnormalities. In addition, the frequencies of non-clonal chromosome aberrations, an indicator of continuous chromosomal instability, in both shp16-hTERT-immortalized cell lines were significantly lower than those in HPV16-E6E7-immortalzed epithelial cell lines (*P* < 0.05) (Supplementary Table S1). These results indicated that the shp16-hTERT-immortalized cervical epithelial cell lines had lower degrees of genetic instability than HPV16-E6E7-immortalzed counterparts.

**Table 2:** Karyotype descriptions of shp16-hTERT -immortalized cervical epithelial cell lines in comparison with HPV16-E6E7-immortalized counterparts

Cell line (PD)	Karyotype*
NC104-shp16-hTERT (PD 30)	46,XX41]/47,XX,+20[12]
NC105-shp16-hTERT (PD 30)	46,XX[36]/47,XX,+7[15]
NC104-shp16-hTERT (PD80)	47,XX,+20[40]/94,XXXX,+20,+20[8]
NC105-shp16-hTERT (PD80)	48,XX,+7,+i(8)(q10),der(17)t(5;17)(?q2;p12)[43]/96,XXXX,+7,+7,+i(8)(q10)×2,der(17)t(5;17)(?q2;p12)×2[4]
NC104-E6E7 (PD80)	46,XX,der(20)dup(20)(q?)t(3;20)(q25;q?)[7]/45∼48,XX,−2,+5,+7,der(20)dup(20)(q?)t(3;20)(q25;q?)[cp11]/89∼94,XXXX,−2,+5,+6,+7,+8,der(20)dup(20)(q?)t(3;20)(q25;q?)×2,−22[cp32]
NC105-E6E7 (PD80)	54∼57,XX,+1,+2,+5,+5,+6,+7,del(7)(q11),der(8;15)(q10;q10),+9,+13,+18,+20,+20,+21[cp50]

* The numbers in square brackets indicate cell numbers identified. “cp” in square brackets means composite karyotypes: no any cell had all listed aberrations.

### DNA damage response

**Figure 5:**
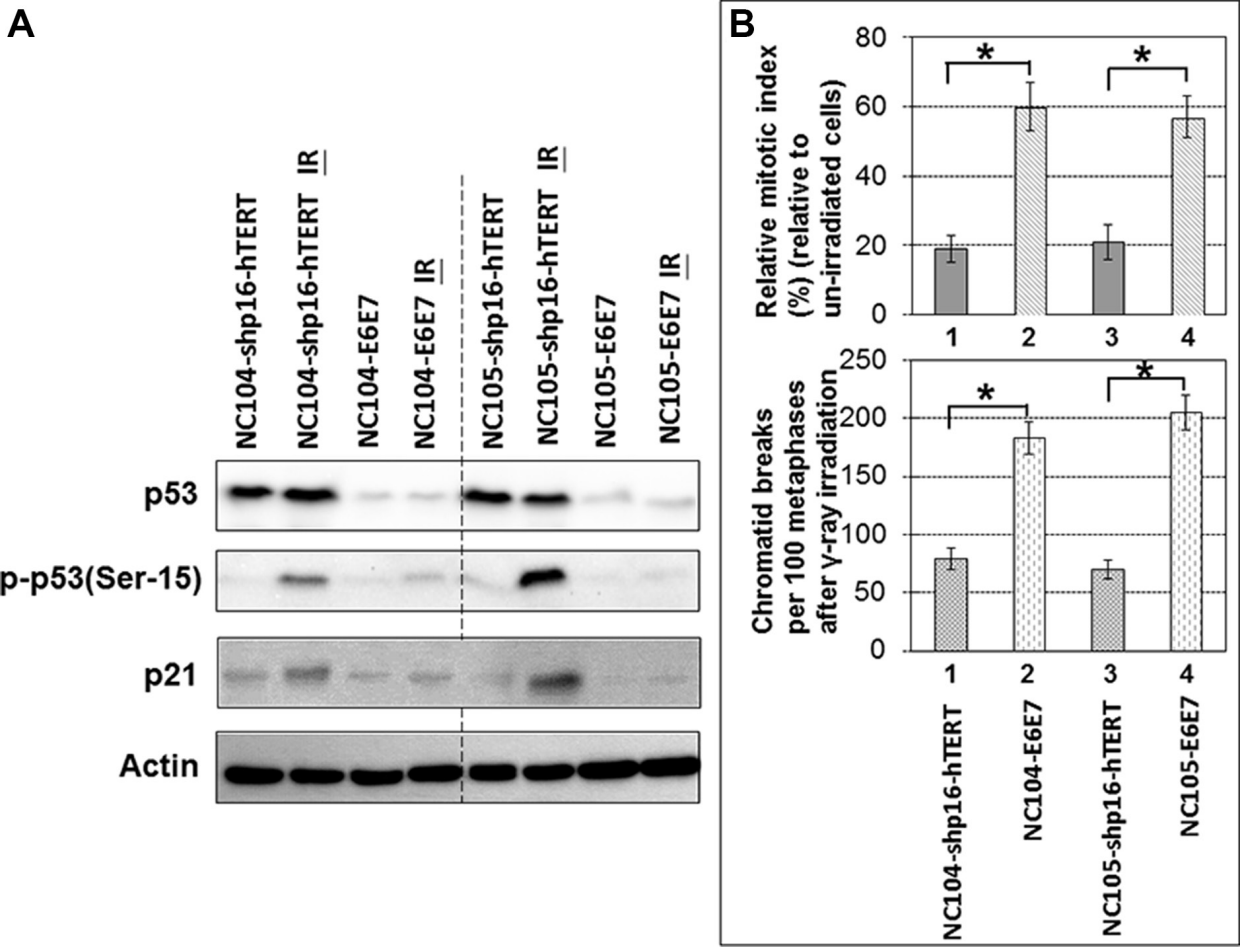
Response of p53 and p21 to ionizing radiation and G2 checkpoint function analysis in shp16-hTERT-immortalized and HPV16-E6E7-immortalized cervical epithelial cell lines (**A**) Western blotting analysis of p53, phospho-53 and p21 in immortalized cervical epithelial cell lines 2 hr after 0.5 Gy γ-ray irradiation. (**B**) Upper chart: Relative mitotic indices in immortalized cervical epithelial cell lines 2 hr after 0.5 Gy γ-ray irradiation (4,000 cells were counted); Lower chart: Frequencies of chromatid breaks in immortalized cervical epithelial cell lines 2 hr after 0.5 Gy γ-ray irradiation (100 metaphases were analyzed). Error bars indicate standard deviations. *P < 0.05.

One of sensitive and pivotal responses to DNA damage is the activation of p53/p21 pathway, which plays important roles in tumor suppression and is thus frequently inactivated in many types of cancer [5]. We therefore examined the response of p53 and p21 to DNA damage in the shp16-hTERT-immortalized cervical epithelial cell lines and compared with that in HPV16-E6E7-immortalized cell lines. We used a low dose (0.5 Gy) of γ-ray irradiation to examine if a sensitive differential response to a low level of DNA damage could be detected. Two hours after irradiation, the expression levels of phosphorylated p53 (at Ser-15) (the active form of p53) and p21 (the downstream target of p53 activation) showed remarkable increase in both shp16-hTERT-immortalized cell lines, although there were some differences between the two cell lines in the increased levels of two proteins (for unknown reasons) (Figure 5A). No obvious change in the total levels of p53 was detected (Figure 5A), indicating that phosphorylated p53(Ser-15) and p21 are more sensitive responders to DNA damage than total levels of p53. It is possible that the high basal levels of total p53 expression in cervical epithelial cells might obscure the detection of subtle changes in total levels of p53 expression in response to a low level of DNA damage. For comparison, HPV16-E6E7-immortalzed cell lines showed barely detectable increase in the expression levels of phosphorylated p53 and p21 two hours after the γ-ray irradiation. These results together demonstrated that the shp16-hTERT-immortalized cervical epithelial cell lines maintained sensitive responses to DNA damage.

### Function of G2 checkpoint

G2 checkpoint is essential for the maintenance of genomic integrity. Previous studies revealed that the expression of HPV16-E6E7 in human fibroblasts and foreskin keratinocytes induced deficiency in G2 checkpoint function [36, 37]. Here we utilized the unique opportunity of comparing the immortalized HPV oncogene-negative and -positive cervical epithelial cell lines derived from the same parental cell sources to examine the differential G2 checkpoint function. If the cells have functional G2 checkpoint, the G2 phase cells will be arrested at G2 phase after DNA damage, while mitotic cells are relatively insensitive to DNA damage thus will continue exiting from mitosis after low doses (< 1 Gy) of γ-ray irradiation, leading to the decrease in mitotic index (percentage of mitotic cells) several hours after γ-ray irradiation. The mitotic cells could be accurately identified by individual chromosome condensation using chromosome spreading technique. The proficiency of G2 checkpoint function could be readily analyzed by the ratio of mitotic index 2 – 3 hr after γ-ray irradiation relative to the mitotic index of un-irradiated cells. This has been termed as relative mitotic index [38–40]. A lower relative mitotic index indicates more stringent G2 checkpoint function. By using this method, we found that the shp16-hTERT-immortalized cell lines exhibited more stringent G2 checkpoint function as evidenced by the significantly lower relative mitotic indices than HPV16-E6E7-immortalized cell lines 2 hr after 0.5 Gy γ-ray irradiation (*P* < 0.05) (Figure 5B). Metaphase cells that escape from defective G2 checkpoint usually contain chromatid breaks [39, 40]. As shown in Figure 5B, both shp16-hTERT-immortalized cervical epithelial cell lines exhibited significantly lower frequencies of chromatid breaks than HPV16-E6E7-immortalized cell lines 2 hr after 0.5 Gy γ-ray irradiation. These results demonstrated that the shp16-hTERT-immortalized cell lines had more stringent G2 checkpoint than HPV16-E6E7-immortalized cell lines.

### Response to replicative stress

**Figure 6:**
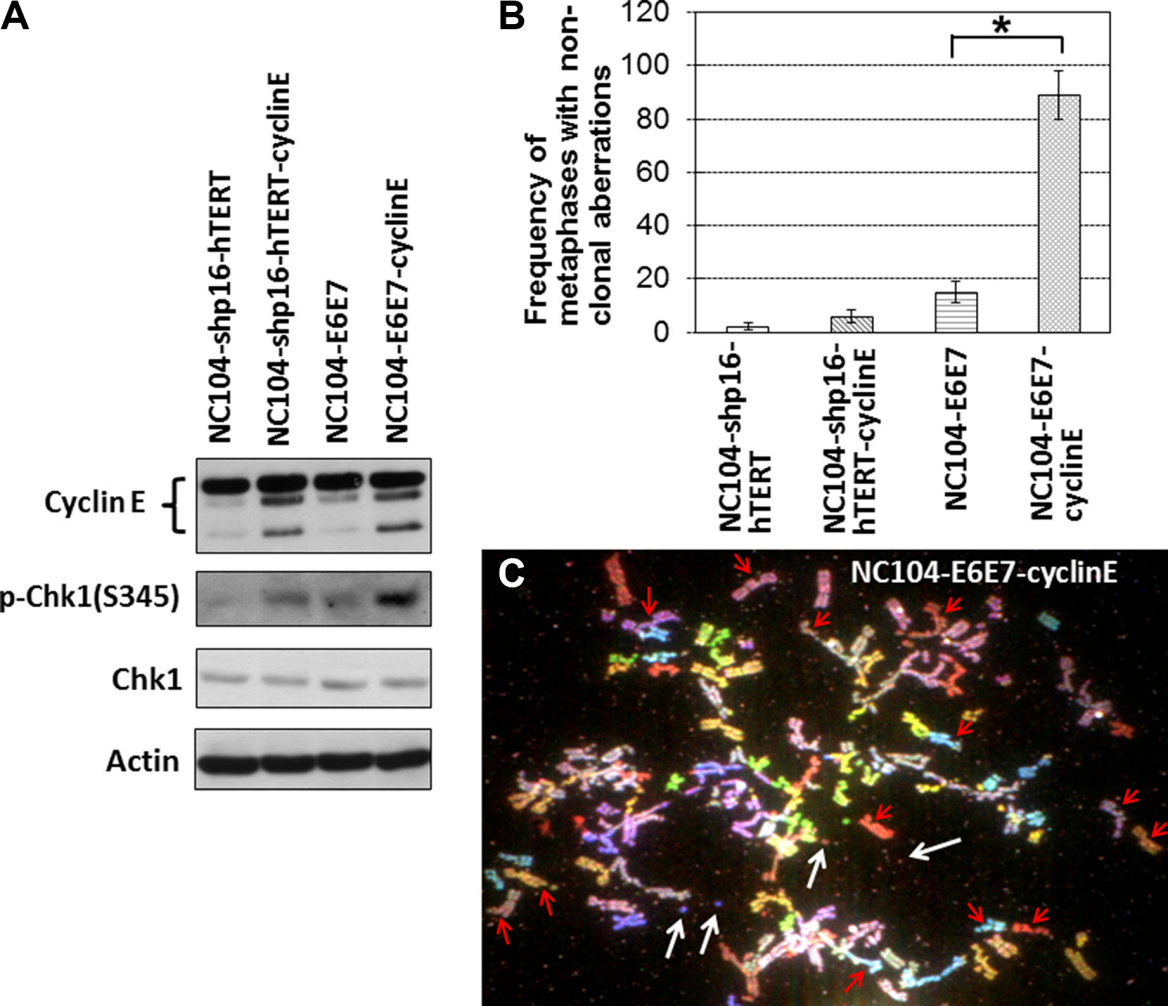
Effects of cyclin E overexpression in immortalized cervical epithelial cells (**A**) Western blotting analysis of cyclin E, Chk1, and phospho-Chk1(S345). Actin serves as protein loading control. (**B**) Frequencies of non-clonal chromosome aberrations in immortalized cervical epithelial cells with cyclin E overexpression or without cyclin E overexpression (infected with retroviruses expressing empty vectors) (100 metaphases were analyzed). Error bars indicate standard deviations. *P < 0.05. (**C**) An example of NC104-E6E7-cyclin E metaphases containing numerous chromosome aberrations. Red arrows indicate some of the chromatid breaks. White arrows indicate examples of minutes. Note that many other chromatid breaks and minutes are visible.

Replication stress-induced chromosomal instability has been proposed to be one of the basic mechanisms for the induction of genetic alterations in cancers [41, 42]. It has been established that the proto-oncogene cyclin E overexpression is a potent inducer of replicative stress [42, 43]. To characterize the differences between shp16-hTERT-immortalized and HPV16-E6E7-immortalized cervical epithelial cells in response to replicative stress, NC104-shp15-hTERT cells and NC104-E6E7 cells at PD 80 were infected with retroviral vectors expressing cyclin E or empty vectors. The successful overexpression of cyclin E was confirmed by Western Blotting analysis (Figure 6A) in both cell lines. A pioneer study showed that cyclin E overexpression induced dramatic chromosome structural aberrations in HPV16-E6E7-expressing human fibroblasts while barely induced detectable structural aberrations in normal fibroblasts [43]. Here we analyzed the metaphases from cyclin-E-overexpressing cervical epithelial cells and the control cells (infected with empty vectors) for detailed chromosome aberration analysis using 24-color SKY. Our results (Figure 6B) showed that the frequency of metaphases containing non-clonal chromosome abnormalities (mainly chromatid breaks and fusions) dramatically increased after cyclin E overexpression in NC104-E6E7 cells, whereas a relatively small extent of increase (*P* > 0.01) was detected in NC104-shp16-hTERT cells after cyclin E overexpression. Figure 6C shows an example of NC104-E6E7 metaphase with numerous chromosome breaks and minutes after cyclin E overexpression. Such complex chromosome aberrations were not found in NC104-shp16-hTERT cells after cyclin E overexpression. In line with the chromosome aberration analysis results, the expression level of phosphorylated Chk1, a classical and sensitive response to replicative stress [42, 44], showed a slight increase in NC104-shp15-hTERT cells while a remarkable increase in NC104-E6E7 cells after cyclin E overexpression (Figure 6A). These results revealed that the shp16-hTERT-immortalized cervical epithelial cells were more resistant to replication stress-induced genetic instability compared with the counterparts immortalized by HPV16-E6E7.

### Transformation phenotype analysis

Analysis of transformation phenotype is important for characterization of immortalized cell lines. The NC104-shp16-TERT, NC105-shp16-hTERT, and their HPV16-E6E7-immortalized counterparts, NC104-E6E7 and NC105-E6E7 cells, were examined for transformation phenotypes at PD 100 by both *in vitro* soft agar colony formation assay and *in vivo* tumorigenicity assay. We found that none of the immortalized cell lines were able to form colonies in soft agar (Supplementary Figure S4), demonstrating that the immortalized cell lines did not acquire anchorage-independent growth ability, which is a crucial characteristic of transformed phenotype. As the positive control, HeLa cells readily formed colonies in soft agar (Supplementary Figure S4). The shp16-hTERT-immortalized and E6E7-immortalized cells were injected into nude mice to monitor tumorigenicity. All four cell lines failed to form tumors in nude mice after 4 months observation, whereas HeLa cells showed tumor formation in nude mice by week 3. These results together showed that the immortalized cell lines did not show transformed phenotype.

## DISCUSSION

There are several novelties in this study. First, to our knowledge, this is the first report on the immortalization of HPV-negative cervical epithelial cell lines without culturing with feeder fibroblasts.

Second, we found that, in contrast to many other cell types (including those reported by our laboratories) that can be immortalized by hTERT overexpression alone after spontaneous selection of cells with loss or expression of a low level of p16^INK4a^ [13, 15, 18–28], cervical epithelial cells maintain the capacity of extremely stringent activation of p16^INK4a^. It is worthwhile to emphasize that we have been continuously culturing hTERT-expressing cervical epithelial cells for the last 5 years and have attempted a total of 83 cultures of 4 cell strains. However, none of the cultures resulted in an immortalized cell line. The cells at senescence stage persistently expressed increased levels of p16^INK4a^. This tight maintenance of p16^INK4a^ activation in HPV-negative cervical epithelial cells has not been reported before and may represent a unique intrinsic property of cervical epithelial cells. This property of cervical epithelial cells may shed light on the reason why HPV-negative cervical cancer is rare. We have achieved efficient immortalization of cervical epithelial cells by ectopic knockdown of p16^INK4a^ expression in combination with hTERT overexpression, confirming that p16^INK4a^ activation is indeed a functional barrier to immortalization of cervical epithelial cells. Knockdown of p53 in combination with hTERT overexpression was unable to induce immortalization of cervical epithelial cells, suggesting that p16^INK4a^ inactivation may play a more important role than p53 inactivation in early process of pre-cancer transformation of cervical epithelial cells without HPV oncogenes. Since the mechanisms underlying the development of HPV-negative cervical cancer are currently poorly understood, the newly immortalized cervical cell lines without using HPV oncogenes can serve as valuable pre-malignant cell models in the stepwise illustration of events leading to HPV-negative cervical cancerous transformation. The newly immortalized cervical epithelial cell lines are relevant to the studies on HPV-negative cervical cancer development because most HPV-negative cervical cancer lost or exhibited low levels of p16^INK4a^ expression [45, 46].

Third, the availability of HPV-oncogene-negative and -positive immortalized cervical epithelial cell lines derived from the same original cell sources offered a unique opportunity, for the first time, for head-to-head comparative studies on functions related to genomic stability. The p53/p21 pathway is well-known to play critical roles in maintaining genomic stability. The basal expression levels of total p53, phosphorylated p53 (Ser- 15), and p21 in both shp16-hTERT-immortalized cervical epithelial cell lines were remarkably higher than those in HPV16-E6E7-immortalized counterparts. In addition, a low dose of γ-ray irradiation induced significant activation of p53 (indicated by phosphorylation at Ser-15) and p21 in shp16-hTERT-immortalized cervical epithelial cells whereas slight or undetectable activation of p53 and p21 in HPV16-E6E7-immortalized counterparts (Figure 5A). In addition, both shp16-hTERT-immortalized cervical epithelial cell lines exhibited the proficient G2 checkpoint function, in contrast to the defective G2 checkpoint function in HPV16-E6E7-immortalized cell lines.

Fourth, the examination on replication stress-induced genomic instability in the newly established immortalized cell lines is another novelty in this study. Replication stress-induced genomic instability has been proposed to a basic and important route leading to cancer development [41, 42]. Replication stress *in vivo* can be induced by hyper-DNA replication due to overexpression of S-phase promoting oncogenes such as cyclin E and cyclin A. In this study, we used cyclin E overexpression to induce replication stress. Compared with HPV16-E6E7-immortalized cells, the shp16-hTERT-immortalized cervical epithelial cells showed remarkably lower degree of Chk1 activation (a sensitive indicator of replication stress) after cyclin E overexpression. This could be explained by the finding that the ability of cyclin E to induce replication stress is suppressed by functional p53/21 pathway [43]. In line with this, the shp16-hTERT-immortalized cells exhibited dramatically lower frequencies of chromosome aberrations than HPV16-E6E7-immortalized counterparts after cyclin E overexpression (Figure 6B). These results suggest that the shp16-hTERT-immortalized cervical epithelial cells had a much better defence against replication-stress-induced genomic instability than HPV16-E6E7-immortalized counterparts.

Karyotype analysis showed that shp16-hTERT-immortalized cervical epithelial cell lines had fewer clonal chromosomal abnormalities than respective HPV16-E6E7-immortalized counterparts analyzed at PD80 (Table 2). Although minor near-tetraploid clones derived from the whole-genome duplication of the near-diploidy were detected in both shp16-hTERT-immortalized cervical epithelial cell lines at PD80, the near-tetraploid cells were stably expanded, which was indicated by completely identical karyotypes in the minor clones. Such stability in the expansion of near-tetraploidy was not observed in HPV16-E6E7-immortalzied cell lines, in which no completely identical near-tetraploidy was found. This is indicated by the “composite karyotype” [cp] in Table 2. In NC105-E6E7 cell line which was still near-diploid, no analyzed cells contained the completely identical karyotypes, indicating a high level of genomic instability. Moreover, non-clonal structural and numerical aberrations were detected at much lower frequencies in shp16-hTERT-immortalized cervical epithelial cell lines compared with respective HPV16-E6E7-immortalized counterparts (Supplementary Table S1), further demonstrating that shp16-hTERT-immortalized cervical epithelial cell lines had lower degrees of genomic instability than HPV16-E6E7-immortalized counterparts. Interestingly, the minor clone with trisomy 20 or trisomy 7 observed at PD30 in NC104-shp16-hTERT and NC105-shp16-hTERT cell line, respectively, became dominant at PD80. In NC105-shp16-hTERT cells at PD80, the dominant clone had 8q isochromosome. These chromosomal alterations were also previously observed in multiple other cell types immortalized by non-viral genetic factors [14, 27, 47]. The common findings suggest that those chromosomal alterations may provide a general growth advantage to immortalized cells, although it is unknown if they are definitely required for immortalization.

For tumorigenic transformation, it is known that multiple potent oncogenes are required. As expected, our shp16-hTERT-immortalized and HPV16-E6E7-immortalized cervical epithelial cell lines did not show tumorigenic phenotype, in agreement with most of previous reports of immortalized cell lines tested for transformation properties [e.g., 6–9, 21, 22, 25, 27].

In summary, we have revealed, for the first time, that cervical epithelial cells are different from many other reported cell types in that cervical epithelial cells possess the intrinsic characteristic of extremely stringent activation of p16^INK4^ expression without HPV oncogene expression. This unique property of cervical epithelial cells may shed light on the reasons why HPV-negative cervical cancer is rare. We have successfully immortalized the first set of HPV-negative cervical epithelial cell lines without using feeder fibroblasts by knockdown of p16^INK4a^ in combination with hTERT overexpression. When compared with HPV16-E6E7-immortalized counterparts, the novel HPV-negative immortalized cervical epithelial cells had lower degrees of chromosomal instability, more sensitive response to DNA damage, more stringent G2 checkpoint, were more resistant to replication stress-induced chromosome aberrations. The shp16-hTERT-immortalized and HPV16-E6E7-immortalized cervical epithelial cell lines were non-tumorigenic in nude mice. The newly immortalized cervical epithelial cell lines can be used not only as much-needed HPV-negative non-malignant control models in studies on novel functions of HPV oncogenes but also as starting models that can be genetically manipulated in a stepwise fashion to investigate the roles of defined genetic alterations in the development of HPV-negative cervical cancers. The cell models with defined genetic alterations may be particularly useful in testing preventive or therapeutic drugs that have specificity against proteins encoded by defined altered genes.

## MATERIALS AND METHODS

### Cell culture

The same primary normal cervical epithelial cells (NC104 and NC105) as we previously reported [11] were used to establish novel immortalized cell lines. Our HPV16-E6E7-immortalized cervical epithelial cell lines (NC104-E6E7 and NC105-E6E7) [11] were used for comparative studies. The epithelial cells were cultured in serum-free media as described [11]. No feeder fibroblasts were used for cell culture. HeLa cells were cultured in DMEM supplemented with 10% fetal bovine serum.

### Vectors and viral infection

The lentiviral vectors expressing short hairpin RNA against p16^INK4a^ and the empty vectors were a kind gift from Prof. J Campisi (Lawrence Berkeley National Laboratory, USA) [20]. The retroviral vector pLXIN-hTERT and the empty vectors were kindly provided by Dr. J.C. Barrett (Laboratory of Molecular Carcinogenesis, National Institute of Environmental Health Sciences, USA) [48]. The retroviral vector expressing short hairpin RNA against p53 was kindly provided by Dr. J. Liu (The University of Texas M. D. Anderson Cancer Center, USA) [32]. The retroviral vector pBabe-Cyclin E and the empty vectors were kindly provide by Dr. Bruce E Clurman (University of Washington School of Medicine Seattle, USA) [43]. The 293T and phoenix-293 cells were used to produce infectious lentiviruses and retroviruses, respectively. The primary cervical epithelial cells at PD 4 were infected with the lentiviruses for p16^INK4a^ knockdown or retroviruses for hTERT overexpression. In addition, the cervical epithelial cells after stable p16^INK4a^ knockdown were re-infected at PD 12 with retroviruses expressing hTERT. Stable gene expression or knockdown was achieved by appropriate drug selection.

### Spectral karyotyping (SKY)

Metaphases were harvested and chromosome spreads prepared as described [11]. Fifty to 100 metaphases were analyzed using SKY for chromosome aberrations as previously reported [49]. The International System for Human Cytogenetic Nomenclature (1995) was applied for karyotype description.

### Western Blotting analysis

For each sample, 20 microgram protein from whole-cell extracts was loaded for Western blotting analysis as described [27]. The primary antibodies against p16^INK4a^ (Ab4-JC2) was from Neomarkers (Fremont, CA); antibodies against Rb, p21, cyclin E, hTERT and actin were from Santa Cruz Biotechnology (Santa Cruz, CA); anti-p53 was from DakoCytomation (Glostrup, Denmark); anti-cyclin D1 (DCS-6) was from BD PharMingen (San Jose, CA); antibodies against phospho-p53(ser-15), CHK1 and phospho-CHK1(ser-345) were from Cell Signaling Technology (Danvers, MA); antibodies against cytokeratins 18 and 19 were from Novocastra Laboratories Newcastle, UK); antibodies against cytokeratin cytokeratins 5/8 and 13 were from Chemicon (Temecula, CA).

### Reverse transcription (RT)-PCR for HPV16 E6 and E7

Qiagen RNeasy Mini Kit (Qiagen, Hilden, Germany) was used for total RNA extraction according to the manufacture's instruction. RT-PCR analysis for HPV16-E6 and E7 was performed as reported [11].

### Telomerase activity measurement

Telomerase activity was analyzed by using TeloTAGGG Telomerase PCR ELISA kits purchased from Sigma-Aldrich (St. Louis, MO). The quantification of telomerase activity (relative to “low activity” controls provided by the manufacturer) was carried out by following the manufacturer's protocols. One microgram of extracted protein was used for each sample.

### Irradiation

One day before irradiation, the culture media were refreshed, and the cells were allowed to reach about 80% confluence. The γ-ray irradiation was carried out at a dose rate of 10 Gy/min in a GammaCell 220 irradiator containing a ^137^Cs radiation source (Atomic Energy of Canada Ltd).

### Chromosome spreading for mitotic index analysis

Cells were harvested without any microtubule inhibitors. Chromosome spreads were prepared using our published protocols [50]. Cells with distinguishable individual chromosome spreads were identified as mitotic cells. For each experimental point, 4,000 cells were counted for the frequency of mitotic cells.

### Soft-agar colony formation assay and tumorigenicity in nude mice

For soft-agar colony formation assay, 5 × 10^4^ cells were plated onto 6-well plate with 0.3% Bacto-agar with serum-free media above a solidified layer of 0.6% agar-base of the same mixture. Colonies were counted as described [27]. For tumorigenicity assay in nude mice, 10^7^ cells were resuspended in the 0.1 ml 1:1 mixture of growth media and Matrigel™ Basement Membrane Matrix (BD Biosciences) for subcutaneous injection into the flank of 6–8 week-old nude mice. The growth of tumor was monitored weekly for up to 4 months after injection.

### Statistical analysis

Statistical differences were analyzed using the two-tailed *T*-test. *P* values < 0.05 were regarded as significant. Error bars in all bar graphs represent standard deviations.

## Supplementary Materials


