# Supplementary material for: Extremely stringent activation of p16INK4a prevents immortalization of uterine cervical epithelial cells without human papillomavirus oncogene expression
Source: Oncotarget. 2016 Jun 17;7(29):45656–70. doi: 10.18632/oncotarget.10120 (PMC5216750; doi:10.18632/oncotarget.10120)
Supplement: Supplementary file 1 [file oncotarget-07-45656-s001.pdf]

Extremely stringent activation of p16^{INK4a} prevents immortalization of uterine cervical epithelial cells without human papillomavirus oncogene expression

Supplementary Materials

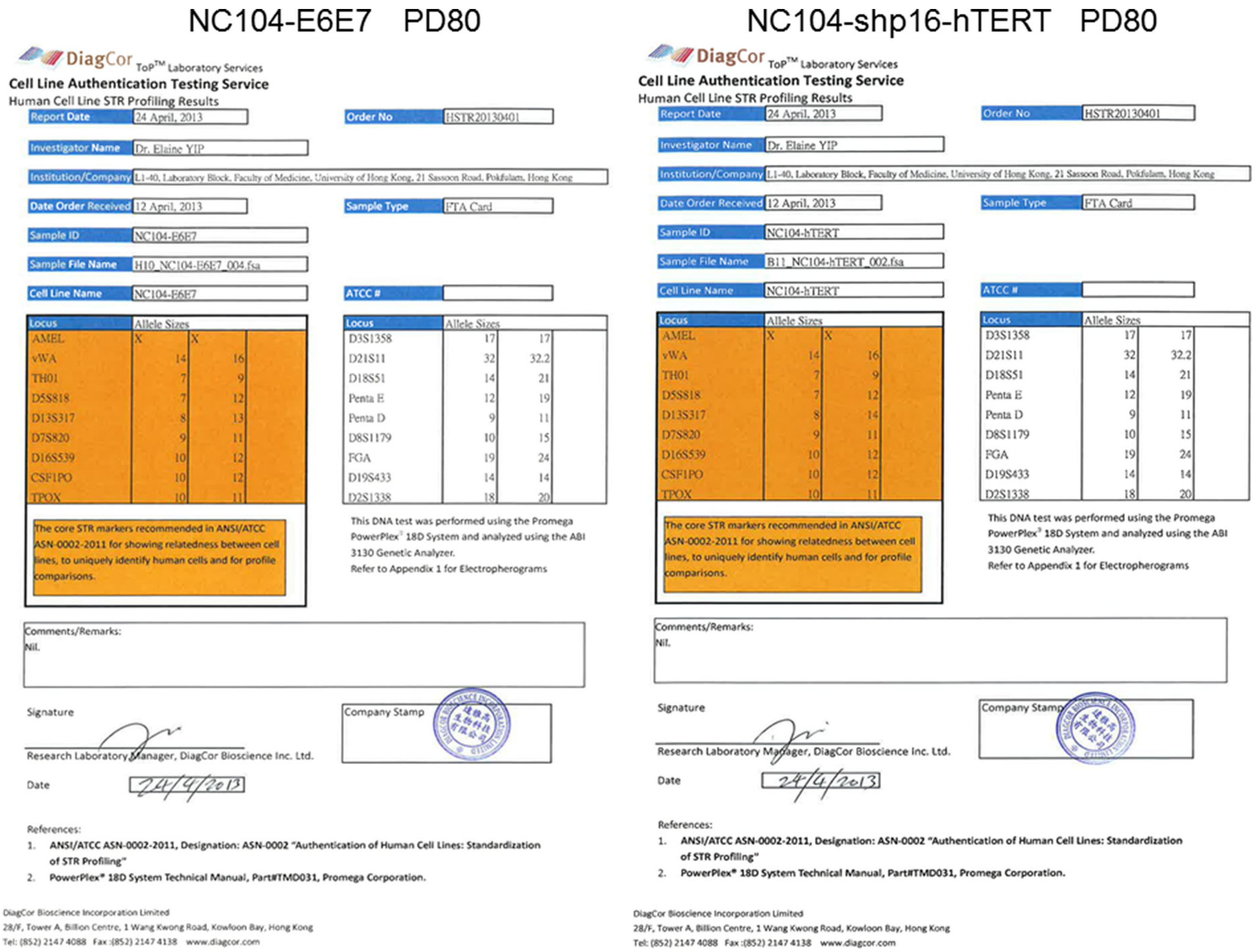

NC104-E6E7 PD80

DiagCor ToP™ Laboratory Services
Cell Line Authentication Testing Service
Human Cell Line STR Profiling Results

Report Date: 24 April, 2013
Order No: HSTR20130401
Investigator Name: Dr. Elaine YIP
Institution/Company: L1-40, Laboratory Block, Faculty of Medicine, University of Hong Kong, 21 Sassoon Road, Pokfulam, Hong Kong
Date Order Received: 12 April, 2013
Sample Type: FTA Card
Sample ID: NC104-E6E7
Sample File Name: H10_NC104-E6E7_004.fsa
Cell Line Name: NC104-E6E7
ATCC #:

Locus	Allele Sizes		
AMEL	X	X	
vWA	14	16	
TH01	7	9	
D5S818	7	12	
D13S317	8	13	
D7S820	9	11	
D16S539	10	12	
CSF1PO	10	12	
TPOX	10	11	

The core STR markers recommended in ANSI/ATCC ASN-0002-2011 for showing relatedness between cell lines, to uniquely identify human cells and for profile comparisons.

Locus	Allele Sizes		
D3S1358	17	17	
D21S11	32	32.2	
D18S51	14	21	
Penta E	12	19	
Penta D	9	11	
D8S1179	10	15	
FGA	19	24	
D19S433	14	14	
D2S1338	18	20	

This DNA test was performed using the Promega PowerPlex® 18D System and analyzed using the ABI 3130 Genetic Analyzer.
Refer to Appendix 1 for Electropherograms

Comments/Remarks:
Nil.

Signature
Research Laboratory Manager, DiagCor Bioscience Inc. Ltd.
Date 24/4/2013
Company Stamp

References:
1. ANSI/ATCC ASN-0002-2011, Designation: ASN-0002 "Authentication of Human Cell Lines: Standardization of STR Profiling"
2. PowerPlex® 18D System Technical Manual, Part#TMD031, Promega Corporation.

DiagCor Bioscience Incorporation Limited
28/F, Tower A, Billion Centre, 1 Wang Kwong Road, Kowloon Bay, Hong Kong
Tel: (852) 2147 4088 Fax :(852) 2147 4138 www.diagcor.com

NC104-shp16-hTERT PD80

DiagCor ToP™ Laboratory Services
Cell Line Authentication Testing Service
Human Cell Line STR Profiling Results

Report Date: 24 April, 2013
Order No: HSTR20130401
Investigator Name: Dr. Elaine YIP
Institution/Company: L1-40, Laboratory Block, Faculty of Medicine, University of Hong Kong, 21 Sassoon Road, Pokfulam, Hong Kong
Date Order Received: 12 April, 2013
Sample Type: FTA Card
Sample ID: NC104-hTERT
Sample File Name: B11_NC104-hTERT_002.fsa
Cell Line Name: NC104-hTERT
ATCC #:

Locus	Allele Sizes		
AMEL	X	X	
vWA	14	16	
TH01	7	9	
D5S818	7	12	
D13S317	8	14	
D7S820	9	11	
D16S539	10	12	
CSF1PO	10	12	
TPOX	10	11	

The core STR markers recommended in ANSI/ATCC ASN-0002-2011 for showing relatedness between cell lines, to uniquely identify human cells and for profile comparisons.

Locus	Allele Sizes		
D3S1358	17	17	
D21S11	32	32.2	
D18S51	14	21	
Penta E	12	19	
Penta D	9	11	
D8S1179	10	15	
FGA	19	24	
D19S433	14	14	
D2S1338	18	20	

This DNA test was performed using the Promega PowerPlex® 18D System and analyzed using the ABI 3130 Genetic Analyzer.
Refer to Appendix 1 for Electropherograms

Comments/Remarks:
Nil.

Signature
Research Laboratory Manager, DiagCor Bioscience Inc. Ltd.
Date 24/4/2013
Company Stamp

References:
1. ANSI/ATCC ASN-0002-2011, Designation: ASN-0002 "Authentication of Human Cell Lines: Standardization of STR Profiling"
2. PowerPlex® 18D System Technical Manual, Part#TMD031, Promega Corporation.

DiagCor Bioscience Incorporation Limited
28/F, Tower A, Billion Centre, 1 Wang Kwong Road, Kowloon Bay, Hong Kong
Tel: (852) 2147 4088 Fax :(852) 2147 4138 www.diagcor.com

Supplementary Figure S1: Results of short tandem repeat (STR) analysis at eighteen loci for NC104-E6E7 and NC104-shp16-hTERT cell lines. Note the identical STR profiles of the two cell lines which were derived from the same cell source.

NC105-E6E7 PD80

DiagCor ToP™ Laboratory Services

Cell Line Authentication Testing Service

Human Cell Line STR Profiling Results

Report Date	24 April, 2013	Order No	HSTR20130401
Investigator Name	Dr. Elaine YIP		
Institution/Company	L1-40, Laboratory Block, Faculty of Medicine, University of Hong Kong, 21 Sassoon Road, Pokfulam, Hong Kong		
Date Order Received	12 April, 2013	Sample Type	FTA Card
Sample ID	NC105-E6E7		
Sample File Name	A11_NC105-E6E7_001.fsa		
Cell Line Name	NC105-E6E7	ATCC #	

Locus	Allele Sizes		
AMEL	X	X	
vWA	14	19	
TH01	9	9	
D5S818	11	12	
D13S317	8	11	
D7S820	8	12	
D16S539	11	12	
CSF1PO	10	11	
TPOX	8	11	

The core STR markers recommended in ANSI/ATCC ASN-0002-2011 for showing relatedness between cell lines, to uniquely identify human cells and for profile comparisons.

Locus	Allele Sizes		
D3S1358	15	15	
D21S11	29	30	
D18S51	14	15	
Penta E	5	11	
Penta D	9	11	
D8S1179	10	13	
FGA	19	23	
D19S433	14	15.2	
D2S1338	22	24	

This DNA test was performed using the Promega PowerPlex® 18D System and analyzed using the ABI 3130 Genetic Analyzer.
Refer to Appendix 1 for Electropherograms

Comments/Remarks:
Nil.

Signature

Research Laboratory Manager, DiagCor Bioscience Inc. Ltd.

Company Stamp

Date 24/4/2013

References:
1. ANSI/ATCC ASN-0002-2011, Designation: ASN-0002 "Authentication of Human Cell Lines: Standardization of STR Profiling"
2. PowerPlex® 18D System Technical Manual, Part#TMD031, Promega Corporation.

DiagCor Bioscience Incorporation Limited
28/F, Tower A, Billion Centre, 1 Wang Kwong Road, Kowloon Bay, Hong Kong
Tel: (852) 2147 4088 Fax :(852) 2147 4138 www.diagcor.com

NC105-shp16-hTERT PD80

DiagCor ToP™ Laboratory Services

Cell Line Authentication Testing Service

Human Cell Line STR Profiling Results

Report Date	24 April, 2013	Order No	HSTR20130401
Investigator Name	Dr. Elaine YIP		
Institution/Company	L1-40, Laboratory Block, Faculty of Medicine, University of Hong Kong, 21 Sassoon Road, Pokfulam, Hong Kong		
Date Order Received	12 April, 2013	Sample Type	FTA Card
Sample ID	NC105-hTERT		
Sample File Name	C11_NC105-hTERT_003.fsa		
Cell Line Name	NC105-hTERT	ATCC #	

Locus	Allele Sizes		
AMEL	X	X	
vWA	14	19	
TH01	9	9	
D5S818	11	12	
D13S317	8	11	
D7S820	8	12	
D16S539	11	12	
CSF1PO	10	11	
TPOX	8	11	

The core STR markers recommended in ANSI/ATCC ASN-0002-2011 for showing relatedness between cell lines, to uniquely identify human cells and for profile comparisons.

Locus	Allele Sizes		
D3S1358	15	15	
D21S11	29	30	
D18S51	14	15	
Penta E	5	11	
Penta D	9	11	
D8S1179	10	13	
FGA	19	23	
D19S433	14	15.2	
D2S1338	22	24	

This DNA test was performed using the Promega PowerPlex® 18D System and analyzed using the ABI 3130 Genetic Analyzer.
Refer to Appendix 1 for Electropherograms

Comments/Remarks:
Nil

Signature

Research Laboratory Manager, DiagCor Bioscience Inc. Ltd.

Company Stamp

Date 24/4/2013

References:
1. ANSI/ATCC ASN-0002-2011, Designation: ASN-0002 "Authentication of Human Cell Lines: Standardization of STR Profiling"
2. PowerPlex® 18D System Technical Manual, Part#TMD031, Promega Corporation.

DiagCor Bioscience Incorporation Limited
28/F, Tower A, Billion Centre, 1 Wang Kwong Road, Kowloon Bay, Hong Kong
Tel: (852) 2147 4088 Fax :(852) 2147 4138 www.diagcor.com

Supplementary Figure S2: Results of short tandem repeat (STR) analysis at eighteen loci for NC105-E6E7 and NC105-shp16-hTERT cell lines. Note the identical STR profiles of the two cell lines which were derived from the same cell source.

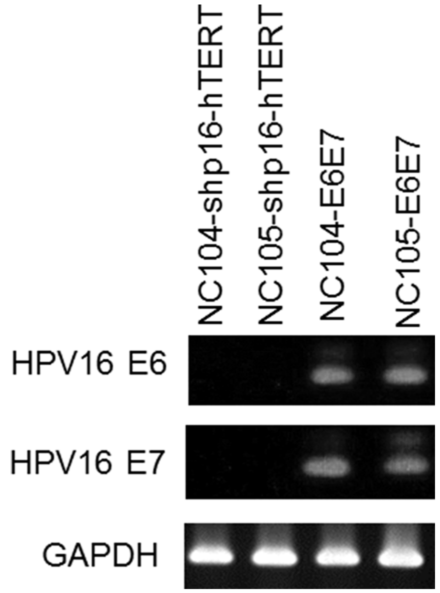

Supplementary Figure S3: RT-PCR for HPV16 E6 and E7 expression. GAPDH served as internal control.

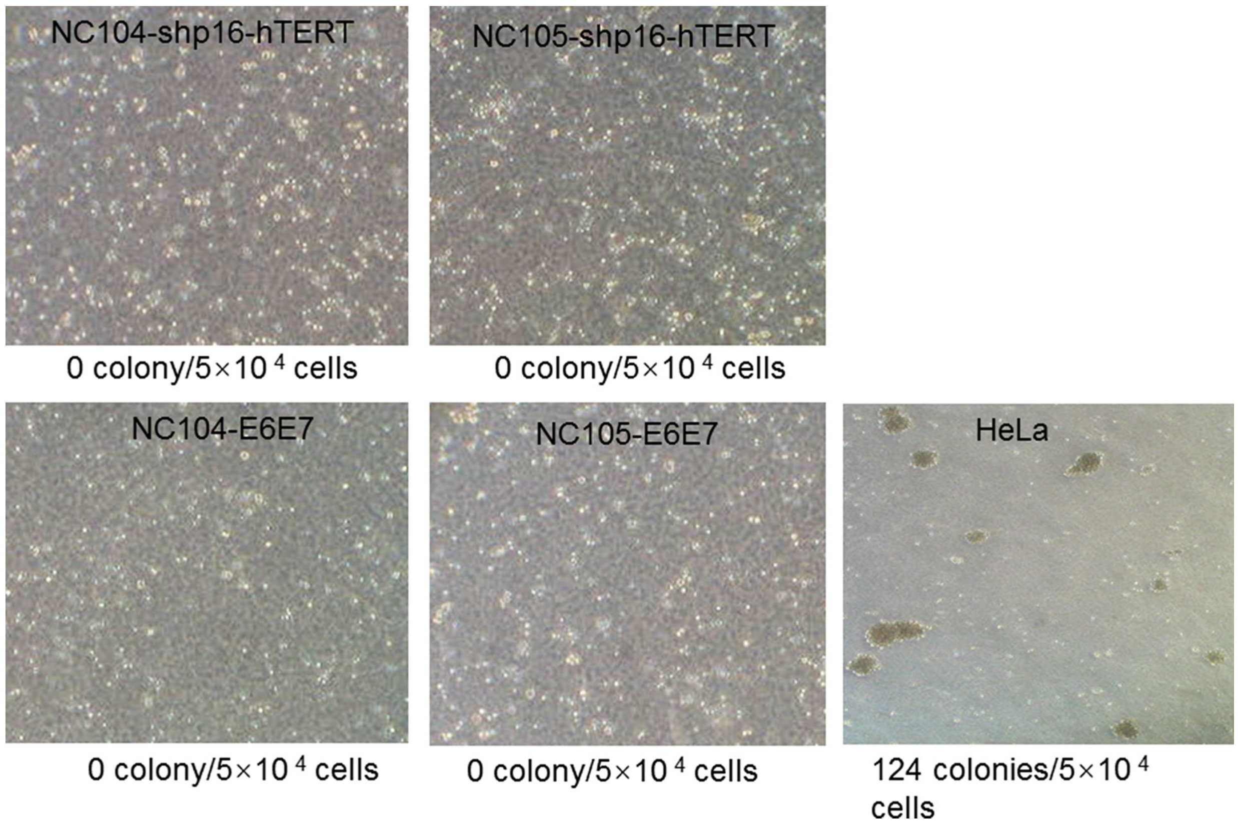

Supplementary Figure S4: Typical images of soft-agar cloning assay.

Supplementary Table S1: Cells with nonclonal chromosomal aberrations in shp16-hTERT-immortalized cervical epithelial cell lines at PD 80 in comparison with HPV16-E6E7-immortalized counterparts (50 metaphases analyzed)

Cell line	Cells with nonclonal translocations	Cells with nonclonal deletions or duplications	Cells with end-fusions	Cells with nonclonal whole-chromosome losses or gains	Cells with pooled forms of nonclonal aberrations
NC104-shp16-hTERT	0	0	0	2	2
NC105-shp16-hTERT	0	0	0	3	3
NC104-E6E7	6	4	3	30	43*
NC105-E6E7	4	3	2	41	50*

*P < 0.5 compared the frequencies of the cells with pooled nonclonal aberrations in respective shp16-hTERT-immortalized cell lines
